# The Relationship between Whole Grain Intake and Body Weight: Results of Meta-Analyses of Observational Studies and Randomized Controlled Trials

**DOI:** 10.3390/nu11061245

**Published:** 2019-05-31

**Authors:** Kevin C. Maki, Orsolya M. Palacios, Katie Koecher, Caleigh M. Sawicki, Kara A. Livingston, Marjorie Bell, Heather Nelson Cortes, Nicola M. McKeown

**Affiliations:** 1 Midwest Biomedical Research/Center for Metabolic and Cardiovascular Health, 211 East Lake Street, Suite 3, Addison, IL 60101, USA; opalacios@mbclinicalresearch.com (O.M.P.); mbell@mbclinicalresearch.com (M.B.); 2 General Mills, Inc., 1 General Mills Blvd., Minneapolis, MN 55426, USA; Katie.Koecher@genmills.com; 3 Nutritional Epidemiology Program, Jean Mayer USDA Human Nutrition Research Center on Aging, Tufts University, 711 Washington St, Boston, MA 02111, USA; csawic01@exchange.tufts.edu (C.M.S.); Kara.Livingston@tufts.edu (K.A.L.); nicola.mckeown@tufts.edu (N.M.M.); 4 Kyzo Nutrition, LLC, 1612 Boulder Ridge Dr., Bolingbrook, IL 60490, USA; hnelsoncortes@gmail.com

**Keywords:** whole grains, body weight, body mass index, body composition, obesity, meta-analysis, randomized controlled trials, prospective cohorts, cross-sectional

## Abstract

Results from some observational studies suggest that higher whole grain (WG) intake is associated with lower risk of weight gain. Ovid Medline was used to conduct a literature search for observational studies and randomized controlled trials (RCTs) assessing WG food intake and weight status in adults. A meta-regression analysis of cross-sectional data from 12 observational studies (136,834 subjects) and a meta-analysis of nine RCTs (973 subjects) was conducted; six prospective cohort publications were qualitatively reviewed. Cross-sectional data meta-regression results indicate a significant, inverse correlation between WG intake and body mass index (BMI): weighted slope, −0.0141 kg/m^2^ per g/day of WG intake (95% confidence interval (CI): −0.0207, −0.0077; r = −0.526, *p* = 0.0001). Prospective cohort results generally showed inverse associations between WG intake and weight change with typical follow-up periods of five to 20 years. RCT meta-analysis results show a nonsignificant pooled standardized effect size of −0.049 kg (95% CI −0.297, 0.199, *p* = 0.698) for mean difference in weight change (WG versus control interventions). Higher WG intake is significantly inversely associated with BMI in observational studies but not RCTs up to 16 weeks in length; RCTs with longer intervention periods are warranted.

## 1. Introduction

Whole grains (WG) are grains that contain the entire nut or seed kernel, including the endosperm, bran, and germ, from the plant from which they are produced [1,2]. WG foods are those that are either 100% whole grain (e.g., WG rolled oats and WG brown rice) or foods that contain some proportion of a whole grain ingredient (e.g., whole grain bread containing whole wheat flour) [1]. WG foods or foods containing significant quantities of WGs tend to be higher in fiber and contain more of other essential nutrients, including iron, zinc, magnesium, selenium, and B vitamins, than refined grains [1]. Data from observational studies consistently indicate a relationship between WG intake and dietary fiber consumption. For example, comparing categories of WG intake in several cohorts in the United States and Europe shows that total dietary fiber intake is significantly associated with WG intake such that total fiber intake is generally 50–100% higher in the top versus the bottom quintile or quartile of whole WG intake [3,4]. WG intake has been associated with healthful eating patterns and lower risk for several morbidities such as cardiovascular disease, diabetes, and obesity [1,5]. The 2015 Dietary Guidelines for Americans (DGA) recommend that at least half of daily grain intake be from WG, and all healthy eating pattern examples, i.e., Healthy U.S.-Style Eating Pattern, Healthy Mediterranean-Style Eating Pattern, and Healthy Vegetarian Eating Pattern, in the DGA report include WG foods [1]. Although daily intakes of total grains are close to recommended amounts, typically, Americans consume excess amounts of refined grains (e.g., white bread, grain-based desserts, white rice, etc.) and do not consume recommended amounts of WG foods (e.g., whole wheat bread, oatmeal, brown rice, etc.) [1].

Findings from observational studies indicate that higher WG intakes are associated with lower risks of weight gain and incident overweight or obesity [6]. In a review by Karl et al. (2012) on the role of WG in body weight regulation, the authors concluded that the studies completed to that point in time had not provided evidence that a hypoenergetic diet that includes 3 to 7 daily servings of WG (48–112 g/day WG) promotes greater weight loss than a control (either no intervention or foods with refined grains) hypoenergetic diet [7]. However, results from some studies have suggested that a hypoenergetic diet including WG-containing foods may be associated with a greater reduction in body fat, particularly abdominal fat, relative to a hypoenergetic, lower WG diet [7,8]. Thus, Pol et al. (2013) concluded that WG consumption does not decrease body weight compared with weight of the control group, but a small beneficial effect on body fat may be present [9].

Many WG foods are good sources of dietary fiber [1,10], and WG intake directly correlates with dietary fiber intake in the U.S. [4]. However, since WG intake among average Americans is <1 serving/day WG [1], and high quantities of fiber-poor refined grains are consumed daily [11], refined grain-based foods are actually the primary source of dietary fiber in the U.S. [11,12]. Incorporation of fiber into the diet, depending on fiber type, can favorably impact health, including attenuation of blood cholesterol and glucose levels, and improved laxation [13,14,15]. Certain types of dietary fibers exert physiological effects that may impact weight status. Beta-glucans and resistant starch type 4, for example, have been found to increase satiety [16,17], though more research is needed. In addition, WG-containing foods collectively contain other bioactive components, such as lignans and phytosterols, shown to exert metabolic effects which have potential to influence body weight and adiposity [18,19,20]. Given that some observational studies report a link between WG intake and body weight, and several WG food components could plausibly affect body weight regulation, the aim of this review was to provide an updated quantitative analysis of data from both observational studies and RCTs examining the relationship of WG intake with body weight status and related variables.

## 2. Methods

### 2.1. Literature Searches

The Preferred Reporting Items for Systematic Reviews and Meta-Analyses guidelines were followed for performing the meta-analyses [21]. A comprehensive literature search was conducted using the Ovid Medline database, which covered studies published from 1946 through January 2018. The search was designed to identify publications of observational studies and RCTs that examined WG intake from WG foods (e.g., oats, quinoa, wild brown rice, etc.) or foods made with WGs (e.g., whole grain breads, whole grain ready-to-eat breakfast cereals, etc.) and not supplements or specific food additives (e.g., dietary fiber supplements). The search strategy used several terms for WG (whole grain, wholegrain, whole-wheat, wild rice, whole rye, buckwheat, oat, etc.). Full search term details are provided in Table S1.

### 2.2. Inclusion and Exclusion Criteria Screening

Inclusion and exclusion criteria were applied through a three-level screening process. Full inclusion and exclusion criteria and details are provided in Table S2 for observational studies and Table S3 for RCTs. Final inclusion criteria included study conducted in humans, English language, intervention arm (for RCTs) or a primary exposure variable (for observational studies) where whole foods (e.g., WG bread, brown rice, etc.) are the source of WG, and the WG-containing food is independently assessable and not part of a mixed intervention such as a diet that increases fruits, vegetables and WGs simultaneously. For the observational database, a weight-based anthropometric outcome of interest (i.e., body weight, body mass index (BMI), adiposity, fat-free mass, waist circumference) had to have been examined. The final exclusion criteria included animal studies, in vitro studies, studies conducted in children (<18 years) or pregnant women, studies assessing gluten-free and/or oral rehydration interventions or associations, reviews, bibliographies, case reports, letters, and/or no WG intervention or assessment.

To identify publications, one scientist (CS) performed two separate literature searches—one aimed at capturing observational studies and another aimed at capturing intervention studies. Publications identified using the search terms underwent the first level of screening using Abstrackr (http://abstrackr.cebm.brown.edu). Abstract screening was conducted by one scientist (KL or CS). Full texts of all publications identified as potentially eligible from the abstract screening phase were then obtained and reviewed for eligibility (level 2 screening) by one scientist (CS or KL); however, texts that were unclear with respect to eligibility were additionally reviewed by an additional scientist (NM). Studies that were excluded for not meeting eligibility criteria during level 2 screening were reviewed in duplicate prior to final exclusion. All discrepancies were resolved by a scientific team with oversight by NM. After full text reviews were completed, PICO (population, intervention, comparator, and outcome) data and results were extracted from eligible publications into one of two databases (one for intervention studies, one for observational studies) that were created with input from the research team. For the intervention study database PICO information was extracted, and then the database was searched for and restricted to studies with anthropometric outcomes of interest for the meta-analysis [22]. The observational database was created later, with the goal of a meta-analysis already established; thus, entries were only included in the database if they had the anthropometric outcomes of interest (per inclusion criteria stated above). All data were extracted and entered into the respective database by one scientist (NM, KL, or CS) and then reviewed in full for accuracy by a second scientist.

Publications in the two databases then underwent an additional level 3 screening by two scientists (OMP and HNC) to determine eligibility for final inclusion in the meta-analyses presented here. Each scientist independently performed the level 3 screening, and disagreements in the final inclusion/exclusion criteria were discussed among the scientific team until consensus was reached. Additional inclusion criteria and exclusion criteria for level 3 screening for observational studies included screening for publications which specifically assessed a measure of weight (kg) or weight status (BMI) as an outcome measure of interest and studies providing cross-sectional data for methodological consistency. Additional inclusion criteria and exclusion criteria for level 3 screening for RCTs screened for studies which specifically assessed a measure of weight (kg) or weight status (BMI) as an outcome variable and where the intervention was at least 12 weeks in length. If additional anthropometric measurements, e.g., whole-body adiposity, waist circumference, fat-free mass, etc., were also part of a study’s outcome assessment for any of the observational studies or the RCTs, baseline data, and in the case of RCTs, end-of-treatment data, were also recorded for potential secondary analyses.

### 2.3. Meta-Regression Analysis of WG Intake: Cross-Sectional Studies

A meta-regression analysis was performed on results of the observational studies to evaluate cross-sectional associations, which applied several assumptions. When mean or median WG intake for each category of WG intake was reported, this value was used in the analysis. When intake was not reported (n = 4 studies from 3 publications) [3,23,24], the midpoint of the range of values reported within a category was employed. For the study reported by Albertson et al. (2016), WG intake was presented in categories consisting of 0 servings/day, >0 to <1 servings/day, or ≥1 serving/day [23]. The >0 to <1 servings/day was estimated to be equivalent to 0.1 g/day to 15.9 g/day and the ≥1 serving/day was estimated to be equivalent to ≥16 g/day. WG intake in the highest category of ≥1 serving/day (≥16 g/day) was further estimated, for this study only, by assuming that approximately 70% of the incremental dietary fiber between the middle and highest WG intake groups was attributable to WG intake, with 3.58 g of fiber per 16 g of WG. Sensitivity analyses were completed to assess the degree to which different assumptions for the Albertson et al. (2016) study impacted the overall results [23]. Varying the estimate for the highest WG intake group from 16 to 40 g/day did not materially alter parameter estimates for the study.

Because of differences in analytical (e.g., statistical, dietary assessment, etc.) methods employed and reporting of multiple analyses over different follow-up periods, some within the same cohorts, it was not possible to conduct a meaningful pooled analysis of data from prospective cohort studies. Therefore, a qualitative assessment of the results was undertaken to evaluate strength, consistency, and dose–response for the associations between baseline WG intake and change in WG intake and change in measures of body weight.

### 2.4. Meta-Regression Analysis of WG Intake: RCTs

The primary outcome of the RCT data meta-analysis was change in body weight (kg), expressed as the standardized mean difference between the exposed group with the highest WG intake reported and the control group. Secondary and sensitivity analyses were conducted to assess the relationship of higher WG versus a control on (1) change in waist circumference (cm), (2) change in body fat percentage, (3) weight change (kg) in a subset of studies that included subjects of both sexes, and (4) weight change (kg) in hypocaloric intervention studies.

Cochrane risk of bias for clinical trials was assessed [25] with nutrition-specific items from a critical appraisal of systematic reviews in the field of nutrition [26]. The methodologic quality of each study was evaluated based on predefined criteria, in accordance with the Agency for Healthcare Research and Quality recommendations for systematic reviews [27]. Study quality for individual domains was determined in duplicate (OMP and HNC), and discrepancies were resolved by consensus in group conference. Preliminary study quality screening included ensuring studies met all the predetermined inclusion and none of the exclusion criteria as well as adequate study length and statistical power for RCTs and the inclusion of relevant cofounding analyses for observational studies. Study quality was assessed in duplicate by two scientists (KCM and OMP) using the Heyland Methodologic Quality Score (MQS)—a tool which rates study methodologic quality on the basis of nine criteria: random assignment, analysis, blinding, patient selection, baseline group comparability, extent of follow-up, treatment protocol, co-interventions, and outcomes [28,29]. Studies are rated between 0 (lowest quality) and 14 (highest quality), and studies with a rating of ≥8 are considered high-quality trials (Table S4).

### 2.5. Statistical Analyses

Descriptive statistics and both unweighted and weighted meta-regression analyses were completed using SPSS Statistics, version 25.0 (IBM, Armonk, NY, USA). Since insufficient data were available for inverse variance weighting for all studies, the weighting scheme used the number of subjects in each group as the weighting factor. Unless otherwise specified, an alpha level of 0.05 was used to define statistical significance.

Pooled analyses were completed using the Meta-analysis with Interactive eXplanations (MIX, version 2.0) program [30]. Within-group changes and standard error (SE) for within-group change were based on reported values obtained from the publication; when these values were not reported, they were calculated from the reported group mean and SE or standard deviation (SD) for the baseline and final values within each group:

SE_Change_ = (SE^2^_Final_ − SE^2^_Baseline_ − 2 × r × SE_Final_ × SE_Baseline_)^0.5^,

where r is the correlation coefficient between baseline and final values (within-group). A value of 0.59 was used for r, as suggested in an empirical evaluation of within-group correlations [31]. The SE of the difference in response between groups was calculated as follows

SE_Difference_ = (SE^2^_ChangeWG_ + SE^2^_ChangeControl_)^0.5^.

Values are reported as standardized mean differences between treatment groups with corresponding 95% confidence intervals (CIs). Pooled estimates with 95% CIs and *p*-values were calculated from random effects meta-analysis models. For the RCT analyses, between-study heterogeneity was assessed with the Q and the *I*^2^ statistics [32].

## 3. Results

**Figure 1:**
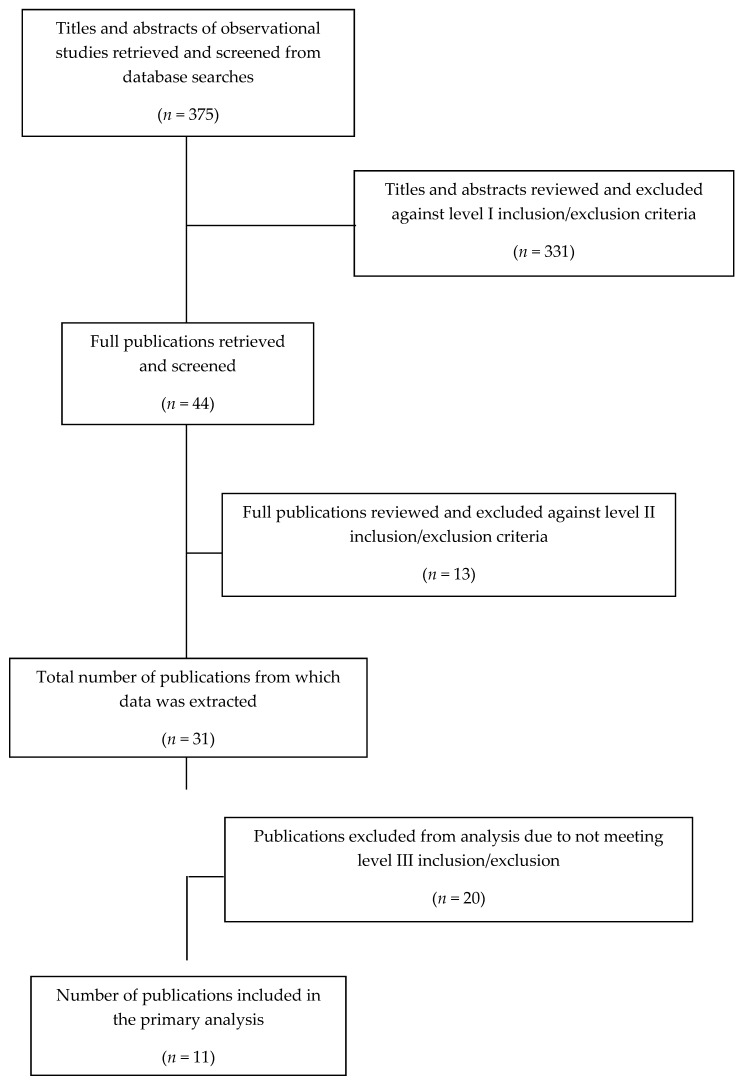
Flow diagram of literature search for observational study analyses inclusion.

**Figure 2:**
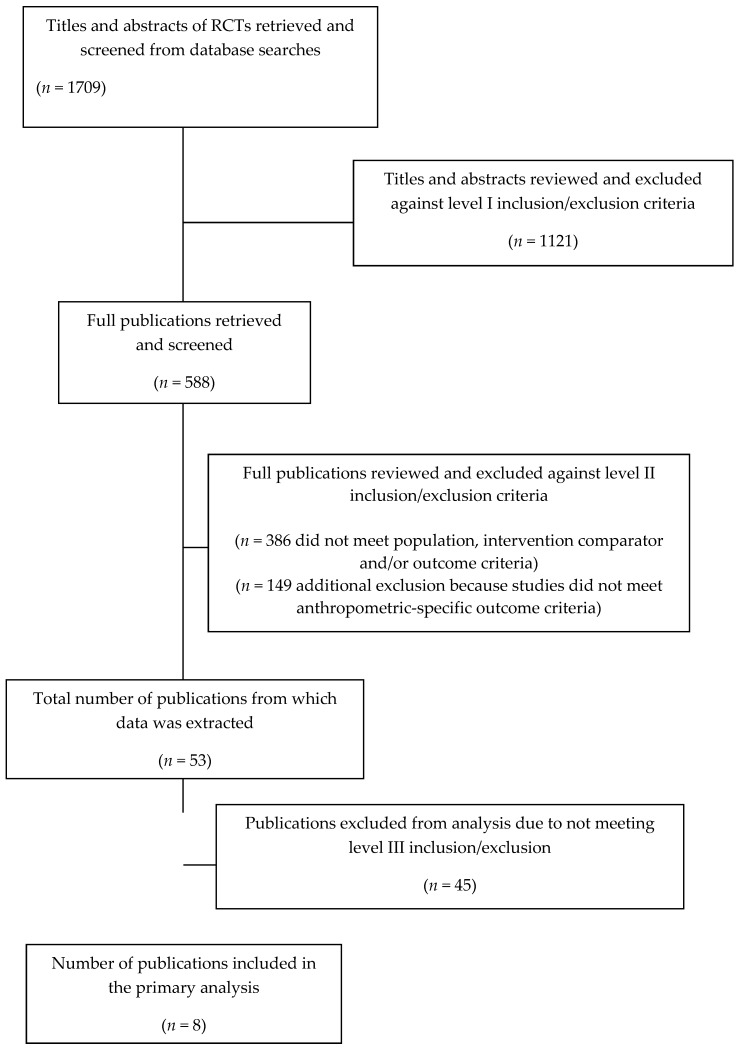
Flow diagram of literature search for RCT analyses inclusion.

A flow diagram summarizing the results of the literature search is shown in Figure 1 for observational studies and Figure 2 for RCTs.

### 3.1. Meta-Regression Analysis: Cross-Sectional Studies

Twelve observational studies—from 11 original publications meeting all the inclusion and none of the exclusion criteria—provided data on WG intake and BMI by WG intake category [3,23,24,33,34,35,36,37,38,39,40]. Of these, nine studies were cross-sectional by design [23,24,33,34,35,36,38,39], and the remaining three were designed as prospective cohort studies but provided cross-sectional data at baseline [3,37,40] (Table S5). The definition of WG varied within these studies, ranging from the inclusion of only a specific food, WG bread [3], or WG definition for foods (foods containing ≥ 51% WG content by weight) [40] to total servings per day as part of the entire diet [23,24,35].

**Figure 3:**
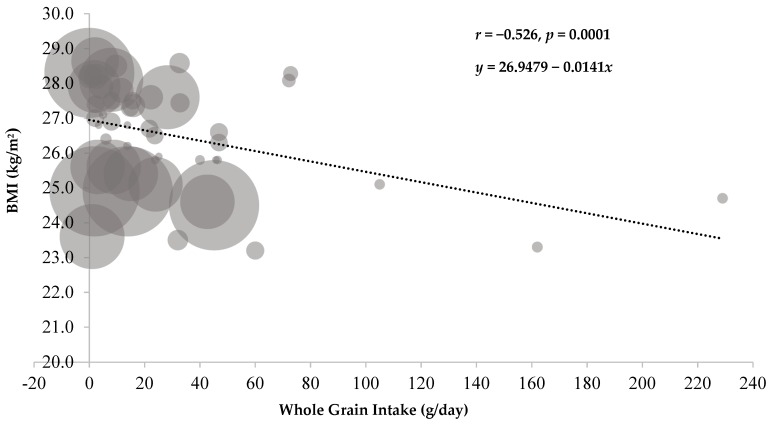
Weighted bubble plot of cross-sectional data from observational studies for the relationship between whole grain intake and body mass index (BMI) ^1^. ^1^ For the unweighted analysis, the regression equation is y = 26.9211 – 0.0146*x*; r = −0.406 (*p* = 0.0034).

The meta-regression analysis of cross-sectional data from the observational studies indicates a significant, inverse association between WG intake and BMI (Figure 3). When the association is assessed as BMI on g/day of WG intake, the unweighted analysis showed a slope of −0.0146 kg/m^2^ per g/day of WG intake (95% confidence interval (CI): −0.0238, −0.0054; r = −0.406; *p* = 0.0034), whereas the weighted analysis indicates a similar slope of −0.0141 kg/m^2^ per g/day of WG intake (95% CI: −0.0207, −0.0077; r = −0.526; *p* = 0.0001).

### 3.2. Qualitative Analysis: Prospective Cohort Studies

A summary of the results from prospective cohort studies is included in Table 1. Six publications were identified that included analyses from four US cohorts of health professionals (Nurses’ Health Study (NHS) I and II, Physicians’ Health Study (PHS), Health Professionals Follow-up Study (HPFS)) and two European cohorts (Seguimiento Universidad de Navarra (SUN) in Spain and the Northern Sweden Health and Disease Study (NSHD)) [3,37,40,41,42,43].

In five of the six prospective studies assessed, aging was shown to be associated with weight gain [37,40,41,42,43]; thus, results reported on the level of weight gain attenuation rather than weight loss. All analyses adjusted for a variety of potential confounders, and the fully adjusted analysis was used in the primary assessment of effect for each study. Two studies reported on the association between baseline intake of a specific type of WG food and change in weight during follow-up. The SUN investigators reported that higher WG bread intake was not significantly associated with weight change or incident overweight/obesity in 9267 subjects in a Mediterranean cohort (54% women) followed for an average of five years [3]. In the PHS of 17,881 US male physicians, increasing intake of WG breakfast cereal was associated with significantly less weight gain at 8 years after adjustment for covariates (1.55 vs. 1.13 kg for extreme categories, *p* for trend = 0.003) [41]. A similar pattern was observed at the longer follow up period of 13 years (2.18 vs. 1.83 kg for extreme categories, *p* for trend = 0.08), although the dose–response association did not reach the 5% level of statistical significance.

In two prospective studies that analyzed change in WG intake from baseline in relation to change in weight, Liu (2003) and Koh-Banerjee (2004), reported inverse associations in body weight within the NHS I cohort (*p* for trend = <0.0001) and HPFS cohort (*p* for trend = 0.002) [37,40]. Specifically, WG intake in the NHS I cohort was associated with less average weight gain (1.07 kg in the highest quintile of WG intake versus 1.58 kg in the lowest quintile of WG over 2–4 years) [37], and for every 40 g/day intake of WG from foods within the HPFS cohort, weight gain was lower by 0.49 kg over an 8-year follow-up [40]. Mozaffarian and colleagues later reported on the pooled data generated from these cohorts and additional data from NHS II and assessed the relationship between lifestyle factors and weight change over follow-up periods up to 20 years (*n* = 120,877) [43]. They observed that within each 4-year period, average weight gain was 1.52 kg in participants, but in multivariate analyses, each serving per day increase in WG intake was associated with 0.17 kg (95% CI 0.22 to 0.22 kg, *p* < 0.001) less weight gain. Winkvist et al. (2017) reported on 10-year follow-up in a subset of the NSHD cohort, and similar to the results in U.S. cohorts, both men and women gained weight, averaging an increase of 2.5 kg over the study period [42]. Each g/2000 kcal of WG intake was significantly inversely associated in multivariate analyses with BMI change in men (*n* = 7641, beta ± SE = −0.13 ± 0.03, *p* < 0.001), but not in women (*n* = 8354, −0.02 ± 0.03, *p* = 0.55). Thus, age-related weight gain is the norm in these prospective cohort studies, and generally, higher intake of WG foods was associated with attenuation of weight gain.

### 3.3. Meta-Analysis: RCTs

**Figure 4:**
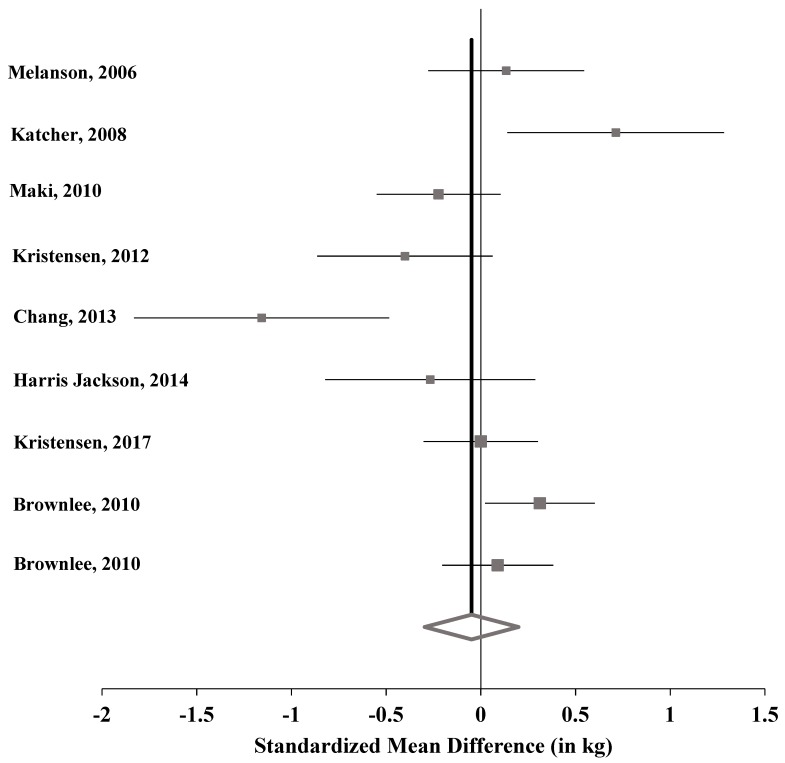
Synthesis forest plot of included studies (values to the left of the line indicate net weight loss for the whole grain intervention).

Data from nine RCTs were extracted from eight publications for the primary analysis and included data from 973 study participants (472 WG intervention, 501 control intervention) [44,45,46,47,48,49,50,51]. Of these, seven were 12-week interventions [44,45,46,47,48,49,50] and two studies were 16-week interventions, but from the same publication [51] (Table S4). WG intake ranged from 32 g/day to 215 g/day for the WG interventions and from 0 g/day to 19 g/day for the studies reporting WG daily intake for the control interventions. Five studies did not report daily WG intake for the control intervention [44,45,46,48,49]. As with the observational data, the definition of WG varied within studies, ranging from the inclusion of only fiber-rich WG breakfast cereals [44] to a diet with only WGs in grain foods [47]. All nine RCTs are considered high-quality trials (Heyland MQS of ≥8). The lowest Heyland MQS score was 9 [45,49], and the highest was 13 [46,47]. As shown in Table 2 and Figure 4, a nonsignificant pooled standardized effect size of −0.049 kg (95% CI −0.297, 0.199, *p* = 0.698) in mean difference in weight change was observed for the WG intervention groups compared with controls. The model showed statistically significant heterogeneity (Q = 28.1, *p* = < 0.001, *I*^2^ = 71.5%). There was no indication of publication bias based on Begg’s and Egger’s test results (data not shown).

Secondary analyses were conducted to assess the effect of WG interventions on change in waist circumference, change in body fat percentage, as well as change in weight in the subsets of RCTs that included both male and female subjects, and in which the dietary interventions were part of a hypocaloric diet (Table 3). The results indicate no significant effect of WG interventions on weight status or other measures for any of the secondary analyses conducted.

## 4. Discussion

The results of the meta-regression analysis of the cross-sectional evidence show a significant inverse relationship between WG intake and BMI. Findings from prospective cohort studies support this relationship, with baseline WG intake and change in WG intake generally showing inverse associations with weight change during follow-up periods of four to 20 years, particularly in the studies with larger numbers of subjects. These relationships remained statistically significant in most cases after adjustment for a variety of covariates and potential confounders. These results are consistent with those from a recent meta-analysis of prospective studies assessing the association of food group intake and risk for overweight/obesity and weight gain [52]. In that analysis, five studies were included in the meta-analysis on incident overweight and/or obesity, which yielded a summary relative risk of 0.85 (95% CI: 0.79–0.91) for high vs. low WG product intake (*I*^2^ = 0%). Three studies were included in that paper’s final dose–response meta-analysis for the WG food group [37,41,53], where an inverse relationship (relative risk for overweight/obesity 0.93, 95% CI 0.89–0.96) per 30 g/day higher intake of WG products was observed. Three studies were included in the analysis of WG product consumption and the risk for weight gain, with weight gain defined as >2 kg during a mean period of 4 years, ≥10 kg during 13 years, or ≥25 kg during an average period of 12 years. The summary relative risk (95% CI) for weight gain was 0.83 (0.70 to 0.97), with *I*^2^ = 16% in the high compared with low intake analysis, and 0.91 (0.82 to 1.02), *I*^2^ = 69%, for each increase of 30 g of whole grain products/day.

Results from the primary and secondary meta-analyses of RCT evidence in the present investigation failed to show a significant effect of higher WG intake on body weight, consistent with findings from some prior reviews [6,9], but not with those from another recent meta-analysis [54]. Reynolds et al. (2019) reported on the results of meta-analysis of 11 RCTs (919 adult participants) assessing the effect of WG on body weight and concluded that WG intake has a significant (mean difference: −0.62 kg, (95% CI −1.19 to −0.05)) association with change in body weight [54]. Some differences exist between the present analyses compared to that of Reynolds et al. (2019). Of the nine studies included in the present meta-analysis and the 11 included in the Reynolds et al. (2019) analysis, only Brownlee et al. (2010) and Chang et al. (2013) were included in both [49,51]. The remaining nine RCTs included by Reynolds et al. (2019) did not meet our inclusion/exclusion criterion of a 12-wk minimum intervention period [55,56,57,58], did not provide sufficient information to quantify the amount of WG in the intervention [59,60], did not provide WG in food (e.g., the intervention was provided as a high-fiber fraction of WG) [61], or instructed subjects to maintain stable body weight [59,62]. Reynolds et al. (2019) excluded trials that employed a hypocaloric (weight loss) diet as part of the intervention, whereas they were included in the present analysis.

One possible explanation for the lack of apparent effect of WG intake on body weight in RCTs in the present analysis may be that WG, per se, may not be causally related to body weight or related anthropometric variables. WG intake could be a marker for lifestyle or habits conducive to lower body weight, and the relationships in observational studies could be attributable to residual confounding. For example, WG intake may correlate with healthful lifestyle factors such as healthy dietary patterns, mindful eating behaviors, greater physical activity levels, and/or longer sleep duration [63,64,65,66]. This phenomenon has been observed in children where oatmeal intake at the breakfast meal was a marker for better overall diet quality and nutrient intake versus other typical breakfast foods (e.g., eggs; ready-to-eat, high-sugar cereals; and pancakes/waffles) [67]. In U.S. adults, WG intake is positively associated with higher diet quality and higher intakes of most micronutrients, dietary fiber, polyunsaturated fatty acids, and total energy, and inversely associated with intake of total and added sugars, monounsaturated fatty acids, saturated fatty acids, and cholesterol [68]. In the United Kingdom, WG intake is associated with higher intakes of magnesium, fiber, and iron, and lower intake of sodium [69]. Therefore, it is possible that the association of WG intake with lower weight status is due to residual confounding and is noncausal [70]. The potential for bias and other types of confounding is an inherent limitation of observational studies, and, ideally, such associations should be confirmed with evidence from well-controlled RCTs [70].

Another possible explanation for the differing relationships in the observational and RCT analyses is that the RCTs may not have been adequate to assess longer-term effects of WG intake on body weight and composition. There are several biologically plausible mechanisms through which higher WG intake could affect energy balance and body composition, including effects on appetite and energy expenditure [7,71]. For example, in a 3-week crossover, blind intervention study assessing the effect of daily breakfast intake of WG rye porridge versus refined flour wheat bread, increases in postprandial subjective ratings of satiety were observed with the rye porridge in healthy adults [71]. In addition, there are some potentially relevant mechanisms that may be mediated by effects of WGs and components, such as fermentable fibers, on gut microbiota [14,72,73,74]. For example, consuming an evening meal containing WG rye flour bread, versus the refined flour, wheat-based bread meal, reduced circulating free fatty acids and increased breath hydrogen, two indicators of increased gut fermentation, in healthy adults [74]. These influences may be too small to have a meaningful impact on the short-term and may require longer periods to manifest. The longest follow-up period in the RCTs assessed was 16 weeks and only two of the nine RCT comparisons were from interventions longer than 12 weeks [51].

An additional consideration is variation in the definitions of WG foods used in both RCTs and observational studies [22]. In 2006, the United States Food and Drug Administration adopted a WG definition that includes intact, ground, cracked, or flaked fruit of grains whose principal components (the starchy endosperm, germ, and bran) are present in the same relative proportions as in the intact grain [22]. Prior to that, some studies included bran and other high dietary fiber foods in their definitions of WG [75]. Since five of the 12 studies included in the observational data for this study are from 2006 or earlier, reported levels of WG intake in those studies potentially include foods/ingredients that are no longer defined as WG. In addition, an analysis of WG intervention study designs found that 73% of WG intervention studies did not specify a definition for the WG product or food, and only 55% of longer-term WG intervention trials reported the amount (as grams or servings) of WG used [22]. With respect to variation in WG exposure within these RCTs, WG-containing foods vary in the quantity of WG within a food or product. A specific threshold of WG intake, or one or more components, may be needed to achieve some physiological effects; for example, thresholds of intake and quality are required to yield adequate viscosity in the stomach with oat intake to affect postprandial glycemia [76]. Furthermore, variations may exist with respect to the type of dietary fiber and other potentially bioactive compounds. For instance, both oats and barley are rich in β-glucans [77], but the major phenolic antioxidants in oats are *p*-hydroxybenzoic acid and vanillic acid, while barley has higher levels of ferulic acid, *p*-coumaric acid, and sinapic acid [77,78].

Lastly, although no significant effects of WGs were observed in RCTs on body composition, the analysis included relatively few studies within which there was marked heterogeneity of results, and results from some trials suggest that body composition and/or body fat distribution may be influenced by WG intake [7,45]. Thus, research with longer intervention periods is needed to assess parameters such as adiposity and waist circumference. Additional RCTs are also needed to assess possible influences of WG intake on the determinants of energy balance (appetite and energy expenditure). Since different WG types likely exert varying physiological effects, RCTs assessing the influences of specific WG types on weight status and related anthropometrics are needed. Additionally, well-controlled RCTs with a clear and standardized definition as to what constitutes a WG food (e.g., ≥51% WG ingredient by weight, only 100% WG food, etc.) are needed to reduce heterogeneity, and exploration of dose–response also warrant further investigation.

## 5. Conclusions

In summary, cross-sectional and prospective data from observational studies suggest an inverse relationship between WG intake and BMI, as well as change in body weight over time. Data from RCTs of at least 12 weeks and up to 16 weeks in duration with WG interventions did not demonstrate significant pooled differences between higher WG and control groups for measures of body weight and related variables such as adiposity and waist circumference. Taken together, the results of these analyses are consistent with dietary recommendations to emphasize intake of WG as part of a healthful eating pattern, although additional research is needed to clarify the potential role of WG food intake in body weight regulation [1].

## Figures and Tables

**Table 1:** Outcomes summary of 6 cohort studies assessing the prospective association between WG intake and weight change ^1^.

Study Author, Year	Cohort (Country)	Subject Number	Follow-Up (years)	WG Exposure	Main Weight Outcome
Liu, 2003 [37]	NHS, females (United States)	74,091	12	Dark bread, WG cereals, popcorn, wheat germ, brown rice, bran, bulgur, kasha, couscous, etc.	WG intake inversely associated with weight gain
Koh-Banerjee, 2004 [40]	HPFS, males (United States)	27,082	8	WG foods with at least 51% WG content by weight	WG intake inversely associated with weight gain
Bazzano, 2005 [41]	PHS, males (United States)	17,881	13	WG ready-to-eat breakfast cereals	WG breakfast cereal intake inversely linked to weight gain
Mozaffarian, 2011 [43]	NHS, NHS II, HPFS (collectively males and females) (United States)	120,877	20	Bran, brown rice, cold breakfast cereal, cooked oatmeal, other cooked breakfast cereal, dark bread, and wheat germ	WG intake inversely associated with the among of weight gain
De la Feuente-Arrillaga, 2014 [3]	SUN Project, males and females (Spain)	9,267	5	WG bread	No association of WG bread intake with weight change
Winkvist, [42] 2017	NSHD, males and females (Sweden)	15,995	10	NR	WG intake inversely associated with BMI change in men only

^1^ Studies included in the review [3,37,40,41,42,43]. Abbreviations: CI: confidence interval; HPFS: Health Professionals Follow-up Study; MD: mean difference; NHS: Nurses’ Health Study; NR: not reported; NSHD: Northern Sweden Health and Disease Study; PHS: Physicians’ Health Study; SUN: Seguimiento Universidad de Navarra; WG: whole grain.

**Table 2:** Random effects meta-analysis model of 9 trials assessing relationship of WG interventions on weight change (kg) ^1,2^.

Study Author, Year	Subjects	SMD	95% CI	*p*-Value	Weight	
Melanson, 2006 [44]	91	0.134	−0.277, 0.545	0.524	11.35%	
Katcher, 2008 [45]	47	0.712	0.140, 1.284	0.015	8.78%	
Maki, 2010 [46]	144	−0.223	−0.550, 0.105	0.183	12.81%	
Kristensen, 2012 [48]	72	−0.401	−0.863, 0.062	0.090	10.47%	
Chang, 2013 [49]	34	−1.158	−1.831, -0.484	0.001	7.43%	
Harris Jackson, 2014 [50]	50	-0.267	−0.822, 0.287	0.345	9.03%	
Kristensen, 2017 [47]	169	0.000	−0.302, 0.302	1.000	13.25%	
Brownlee, 2010 [51]	185	0.312	0.023, 0.601	0.035	13.47%	
Brownlee, 2010 [51]	181	0.089	−0.204, 0.382	0.551	13.41%	
Pooled	973	−0.049	0.199, −0.388	0.698	100.00%	

^1^ Studies included in the analysis are reference numbers [44,45,46,47,48,49,50,51]. ^2^ Heterogeneity: Q = 28.1, *p* = < 0.001, *I*^2^ = 71.5%; Abbreviations: CI: confidence interval; SMD: standardized mean difference.

**Table 3:** Secondary meta-analyses of RCTs assessing the outcome of WG intake (g/day) on waist circumference, body fat percentage, or weight change (kg) ^1,2^.

Secondary Analysis	Included Studies	Subjects	SMD	95% CI	*p*-Value
Waist Circumference	Katcher, 2008 [45] Maki, 2010 [46] Kristensen, 2012 [48] Harris Jackson, 2014 [50] Kristensen, 2017 [47]	482	0.276	−0.436, 0.989	0.447
Body Fat Percentage	Katcher, 2008 [45] Chang, 2013 [49] Harris Jackson, 2014 [50] Kristensen, 2017 [47] Brownlee, 2010 [51] Brownlee, 2010 [51]	666	0.042	−0.573, 0.656	0.895
Mixed Population	Melanson, 2006 [44] Katcher, 2008 [45] Maki, 2010 [46] Chang, 2013 [49] Harris Jackson, 2014 [50] Brownlee, 2010 [51] Brownlee, 2010 [51]	732	−0.016	−0.329, 0.297	0.921
Hypocaloric Diet	Melanson, 2006 [44] Katcher, 2008 [45] Maki, 2010 [46] Kristensen, 2012 [48] Harris Jackson, 2014 [50] Kristensen, 2017 [47]	573	−0.031	−0.291, 0.229	0.814

^1^ Studies included in the secondary analyses [44,45,46,47,48,49,50,51]. ^2^ Weight change was the analysis outcome for the secondary analyses of RCTs employing a mixed (male and female subjects) population or RCTs with a study design which included a hypocaloric diet as part of the interventions. Abbreviations: CI: confidence interval; SMD: standardized mean difference; RCT: randomized controlled trial; WG: whole grains.

## Supplementary Materials

The following are available online at https://www.mdpi.com/2072-6643/11/6/1245/s1, Table S1: Search terms used to identify relevant observational and RCT publications for the meta-regression analyses of WG, Table S2: Inclusion and exclusion criteria for observational studies, Table S3: Inclusion and exclusion criteria for randomized controlled trials, Table S4: Summary of the nine trials included in the meta-analysis of data obtained from RCTs assessing the effect of WG intake (g/day) on body weight (kg), Table S5: Summary of the data obtained from the 12 observational studies included in the meta-regression analysis assessing the association between WG intake (g/day) and weight status (BMI, kg/m^2^),.

## Abbreviations

BMI	body mass index
CI	confidence interval
HPFS	Health Professionals Follow-up Study
MD	mean difference
NHS	Nurses’ Health Study
NSHD	Northern Sweden Health and Disease Study
PHS	Physicians’ Health Study
RCT	randomized controlled trial
RG	refined grain
SD	standard deviation
SE	standard error
SEM	standard error of the mean
SMD	standardized mean difference
SUN	Seguimiento Universidad de Navarra
WG	whole grain
