# Supplementary material for: The Relationship between Whole Grain Intake and Body Weight: Results of Meta-Analyses of Observational Studies and Randomized Controlled Trials
Source: Nutrients. 2019 May 31;11(6):1245. doi: 10.3390/nu11061245 (PMC6627338; doi:10.3390/nu11061245)
Supplement: Supplementary file 1 [file nutrients-11-01245-s001.pdf]

Table S1. Search terms used to identify relevant observational and RCT publications for the meta-regression analyses of WG.

**Terms used to identify all studies**	**Additional terms used to limit identified studies to RCTs**
whole grain* OR wholegrain* OR whole-grain* OR wholemeal OR whole meal OR whole-meal OR wholewheat OR whole wheat OR whole-wheat OR brown rice OR wild rice OR purple rice OR black rice OR red rice OR whole rice OR whole barley OR hulled barley OR hull-less barley OR whole corn OR popcorn OR whole rye OR whole oat* OR oat* OR millet* OR fonio OR sorghum OR milo OR teff OR triticale OR amaranth OR buckwheat OR quinoa OR kaniwa OR canihua OR spelt OR emmer OR faro OR farro OR einkorn OR kamut OR durum OR bulgur OR freekeh OR whole grain cereal* OR wholegrain cereal*	randomized controlled trial OR controlled clinical trial OR randomized OR placebo OR clinical trials as topic OR randomly OR trial OR Random Allocation OR Double-blind Method OR Single-Blind Method OR clinical trial OR placebos OR random$ comparative study OR Evaluation studies OR Cross-Over Studies OR latin square OR intervention studies OR dietary intervention

Abbreviations: RCT, randomized controlled trial; WG, whole grains.

Table S2. Inclusion and exclusion criteria for observational studies.

The specific inclusion criteria will be [1]:

- Original, epidemiological research studies categorized as one of the following:
  - Prospective cohort studies
  - Retrospective cohort studies
  - Case-control studies
  - Cross-sectional studies
- Adult (≥18 y) human subjects
- Whole grain food(s) consumption is a primary exposure of interest
- A clear definition of the whole grain food(s) is provided
- A clear description is included of continuous or categorical assessment(s) of intake
- Documented quantitative (e.g., grams, ounces or serving equivalents per day or week) intake of whole grain food(s) for each population group and per category (if applicable)
- Duration or length of follow-up period of ≥12-weeks for longitudinal studies
  - Based on the European Food Safety Authority guidelines for weight loss claims [2]
- Body weight or a closely related variable such as body mass index is an assessed outcome of interest
- A measure of estimate (e.g., relative risk, odds ratio, hazard ratio, correlation coefficient) for body weight or a closely related variable such as body mass index
- A measure of variability (e.g., confidence interval) for the whole grain intake association to one or more body weight outcomes
  - Measure of estimate (and variabilities) will be recorded, when available, for body mass index, fat mass and/or adiposity
- Publication in the English language
- Adjustment for relevant covariates (e.g. confounding of factors that can affect weight/anthropometric outcomes, such as physical activity, sex, age, smoking status, education, etc.)

The specific exclusion criteria will be [1]:

- Randomized, controlled trials (parallel or crossover design), dietary intervention studies, individual case reports, systematic reviews, meta-analyses, bibliographies, reviews, letters and comments
- Studies in animals
- In vitro studies
- Studies where the whole grain food intake is part of a multicomponent assessment and the association of the whole grain food intake to body weight cannot be isolated (e.g. studies where a dietary pattern that includes whole grains, fruits, and vegetables simultaneously)
- Studies assessing associations related to individual grain components, such as bran or germ or whole grain fiber [such as cereal fiber] or a dietary supplement, and not the entire whole grain food(s).
- Studies in pregnant or lactating women
- Studies in children (<18 y)
- Studies using a weight loss medication, supplement and/or drug therapy
- Studies including subjects with certain chronic diseases such as cancer, diagnosis of cardiovascular disease (e.g., myocardial infarction, stroke, etc.), or chronic kidney disease at baseline
    - NOTE: Studies that included subjects with the chronic conditions of obesity, type 2 diabetes and/or metabolic syndrome will be exempt from the chronic diseases exclusion criterion

Table S3. Inclusion and exclusion criteria for randomized controlled trials.

The specific inclusion criteria will be [1]:

- Randomized, controlled trial (parallel or crossover design)
- Adult (≥18 y) human subjects
- Whole grain food as the main intervention
- Documented quantitative (e.g. grams, ounces or serving equivalents per day or week) intake of whole grain food(s) for each arm
- Intervention exposure duration for each arm ≥ 12-weeks
  - Based on the European Food Safety Authority guidelines for weight loss claims [2]
- Baseline measurements of body weight
- Body weight outcome measurement for each arm
- A measure of variability (SD or SE) of body weight
  - Fat mass and body mass index will be recorded, when baseline measurements, end-of-treatment for each arm measurements and variability measurements collectively are present for these outcomes
- Publication in the English language

The specific exclusion [1] criteria will be:

- Cross-sectional studies, retrospective or prospective cohort studies, or any other observational studies, case-control studies and single-arm studies (interventions with no control group)
- Studies in animals
- In vitro studies
- Trials that specifically required subjects to maintain weight
- Trials where the whole grain intervention is part of a multicomponent intervention and the effect of the whole grain cannot be isolated [e.g. studies where a diet intervention increases consumption of whole grains, fruits, and vegetables simultaneously or intervention testing a whole grain muffin with added fiber versus a refined grain muffin without the added fiber)]
- Trials where the intervention is based only on individual grain components, such as bran or germ or whole grain fiber (such as cereal fiber) or a dietary supplement and not a whole grain food(s)
- Trials where the intervention is given via tube feeding or enteral nutrition
- Trials in pregnant or lactating women
- Trials in children (<18 y)
- Trials using a weight loss medication, supplement and/or drug therapy
- Studies including subjects with certain chronic diseases such as cancer, diagnosis of cardiovascular disease (e.g., angina pectoris), cardiovascular disease event (e.g., myocardial infarction, stroke, etc.), or chronic kidney disease at baseline
  - NOTE: Studies that included subjects with the chronic conditions of obesity, type 2 diabetes and/or metabolic syndrome will be exempt from the chronic disease exclusion criteria

Table S4. Summary of the 9 trials included in the meta-analysis of data obtained from RCTs assessing the effect of WG intake (g/d) on body weight (kg).[1,2.]

			WG Condition		Control Condition		Quality Assessment
**Author, Year**	**Subject Number**	**Duration (days)**	**Treatment Products**	**WG (g/d)**	**Control Products**	**WG (g/d)**	**MQS Score**
Melanson, 2006[3]	91	84	Fiber-rich WG cereals + hypocaloric diet + exercise	32	hypocaloric diet (no cereals) + exercise	NR	11
Katcher, 2008[3]	47	84	WG foods	80	RG foods	NR	9
Maki, 2010[3]	144	84	WG oat RTE cereal	66	Low-fiber breakfast, snacks	NR	13
Kristensen, 2012[4]	72	84	Whole wheat foods (bread, pasta, biscuits)	105	Refined wheat foods (bread, pasta, biscuits)	NR	10
Chang, 2013	34	84	Oatmeal	75	Oat cereal appearance (no beta-glucan)	NR	9
Harris Jackson, 2014[4]	50	84	Variety of WG foods	215	RG only	0	11
Kristensen, 2017[4]	169	84	WG diet (breakfast cereals, bread, pasta, rice, etc.)	124	RG diet	0.5	13
Brownlee, 2010[4]	185	112	Variety of WG foods (oats, cereals, bars, etc.)	74	RG foods	19	11
Brownlee, 2010[4]	181	112	Variety of WG foods (oats, cereals, bars, etc.)	99	RG foods	19	11

[1]Studies included [14-21]. [2]When the control treatment did not provide WG intake amount (NR), an intake of 0g/d was assumed. [3]WG amounts were provided only as servings/d and were converted to g/d using the conversion of 16g = 1 serving and/or calculations from product information provided. [4]WG amounts varied between subjects so a mean/median for the diet condition was used listed. [5]MQS scoring systems ranges from 0 (lowest quality) to 14 (highest quality); a score of ≥ 8 is considered to be a high methodological quality study based on randomization, analysis, blinding, subject selection, baseline group comparability, follow-up, treatment protocol, intervention methodology and outcomes assessment criteria. Abbreviations: MQS, Heyland Methodological Quality Score; NR, not reported; RCT, randomized controlled trial; RG, refined grains; RTE, ready to eat; WG, whole grains.

Table S5. Summary of the data obtained from the 12 observational studies included in the meta-regression analysis assessing the association between WG intake (g/d) and weight status (BMI, kg/m^2).[1.]

Study	Study Design	Subject Number	Covariate Adjustments in Data Used for Meta-Analysis	Definition/Type of WG Exposure	Category Assignment	Midpoint WG Intake (g/d)
**Liu, 2003**	Prospective cohort	74,091	Age, energy intake	Dark bread, WG breakfast cereal (≥25% WG or bran by weight), popcorn, cooked oatmeal, wheat germ, brown rice, bran, bulgur, kasha, couscous	Tertial 1	1.90
					Tertial 2	13.89
					Tertial 3	45.10
**Koh-Banergee, 2004**	Prospective cohort	27,082	Age, energy intake	WG foods with ≥51% WG content by weight	Quintile 1	3.00
					Quintile 2	8.80
					Quintile 3	15.00
					Quintile 4	23.90
					Quintile 5	42.70
**De la Fuente-Arrillaga, 2014**	Prospective cohort	9,267	Age, sex	WG bread	Quartile 1	1.00
					Quartile 2	32.00
					Quartile 3	60.00
					Quartile 4	162.00
**McKeown, 2002**	Cross-sectional	2,941	Age, sex, energy intake, treatment of hypertension, smoking, multivitamin use, estrogen use and physical activity	Any breakfast cereal ≥ 25% WG or bran by weight; other WG foods included dark bread, popcorn, cooked oatmeal, wheat germ, brown rice, and other whole grains	Quintile 1	2.06
					Quintile 2	8.00
					Quintile 3	14.63
					Quintile 4	21.71
					Quintile 5	46.86
**Esmaillzadeh, 2005**	Cross-sectional	827	age, total energy intake, energy from fat, use of blood pressure medication, use of estrogen, smoking, physical activity, meats and fish consumption, fruit and vegetables intake	Dark breads (Sangak, Barbari, Taftoon), barley bread, popcorn, cornflakes (a WG breakfast cereal), wheat germ and bulgur	Quartile 1	6.00
					Quartile 2	40.00
					Quartile 3	105.00
					Quartile 4	229.00
**Sahyoun, 2006**	Cross-sectional	535	age, sex, race, education, marital status, smoking, exercise, BMI, alcohol intake, energy intake, percentage SFA intake and use of antihypertensive or lipid-lowering medication	NR (states that the total number of grain servings was divided into WG and RG on the bases of the proportion of the respective ingredients in the grain food)	Quartile 1	4.96
					Quartile 2	13.76
					Quartile 3	23.84
					Quartile 4	46.40
**Lutsey, 2007**	Cross-sectional	5,496	Age, sex, race, education, survey center, energy intake, current smoking, current alcohol use, intake of fruit, vegetables, refined grains, dairy, fish and poultry, meat, leisure physical activity	WG intake determined by summing servings/d of the following foods: WG breakfast cereal, oatmeal, dark bread, bran muffins, brown or wild rice; WG cereals contained ≥3 g dietary fiber/100g	Quintile 1	0.32
					Quintile 2	2.40
					Quintile 3	4.32
					Quintile 4	11.52

			and sedentariness score		Quintile 5	22.24
**McKeown, 2009**	Cross-sectional	434	Age, sex, total energy intake, percent energy from fat, physical activity, smoking, alcohol intake, and multivitamin use	Breakfast cereals containing ≥ 25% WG by weight	Quartile 1	3.36
					Quartile 2	13.76
					Quartile 3	25.12
					Quartile 4	45.76
**McKeown, 2010**	Cross-sectional	2,834	Age, sex, smoking status, total energy, and alcohol intake	Grains that "consist of the intact, ground, cracked or flaked fruit of the grains, whose principal components—the starchy endosperm, germ and bran—are present in the same relative portions as they exist in the intact grain"	Quintile 1	2.24
					Quintile 2	8.00
					Quintile 3	15.68
					Quintile 4	23.68
					Quintile 5	46.88
**O'Neil, 2010**	Cross-sectional	7,039	Age, energy, gender, ethnicity, cereal fiber	MyPyramind equivalents database for USDA Survey food codes 1994-2002 MPED version 1.0 and 2003-2004 MPED version 2.0	Quartile 1	1.76
					Quartile 2	16.00
					Quartile 3	32.80
					Quartile 4	72.16
**O'Neil, 2010**	Cross-sectional	6,237	Age, energy, gender, ethnicity, cereal fiber	MyPyramind equivalents database for USDA Survey food codes 1994-2002 MPED version 1.0 and 2003-2004 MPED version 2.0	Quartile 1	2.08
					Quartile 2	9.60
					Quartile 3	32.64
					Quartile 4	72.80
**Albertson, 2016**	Cross-sectional	29,638	Age, gender, race/ethnicity, total calorie intake, alcohol intake and physical activity	Grains which included the entire kernel (germ, bran and endosperm), e.g. oatmeal, popcorn, whole wheat, whole barley, wild rice and quinoa	Tertial 1	0.00
					Tertial 2	7.90
					Tertial 3	28.30

[1] Studies included [3-13]. Abbreviations: BMI, body mass index; HRT, hormone replacement therapy; MPED, MyPyramid equivalents database; MUFA, monounsaturated fatty acids; NR, not reported; PUFA, polyunsaturated fatty acids; SFA, saturated fatty acid; WG, whole grains.

References

1. Chen, M.; Pan, A.; Malik, V.S.; Hu, F.B. Effects of dairy intake on body weight and fat: a meta-analysis of randomized controlled trials. *Am J Clin Nutr* **2012**, *96*, 735-747, doi:10.3945/ajcn.112.037119.
2. EFSA Panel on Dietetic Products, Nutrition and Allergies (NDA); Guidance on the scientific requirements for health claims related to appetite ratings, weight management, and blood glucose concentrations. EFSA Journal 2012;10(3):2604. [11 pp.]. Available at: www.efsa.europa.eu/efsajournal Accessed on 06 December 2018.
3. Sahyoun, N.R.; Jacques, P.F.; Zhang, X.L.; Juan, W.; McKeown, N.M. Whole-grain intake is inversely associated with the metabolic syndrome and mortality in older adults. *Am J Clin Nutr* **2006**, *83*, 124-131, doi:10.1093/ajcn/83.1.124.
4. O'Neil, C.E.; Zanovec, M.; Cho, S.S.; Nicklas, T.A. Whole grain and fiber consumption are associated with lower body weight measures in US adults: National Health and Nutrition Examination Survey 1999-2004. *Nutr Res* **2010**, *30*, 815-822, doi:10.1016/j.nutres.2010.10.013.
5. McKeown, N.M.; Meigs, J.B.; Liu, S.; Wilson, P.W.; Jacques, P.F. Whole-grain intake is favorably associated with metabolic risk factors for type 2 diabetes and cardiovascular disease in the Framingham Offspring Study. *Am J Clin Nutr* **2002**, *76*, 390-398, doi:10.1093/ajcn/76.2.390.
6. McKeown, N.M.; Troy, L.M.; Jacques, P.F.; Hoffmann, U.; O'Donnell, C.J.; Fox, C.S. Whole- and refined-grain intakes are differentially associated with abdominal visceral and subcutaneous adiposity in healthy adults: the Framingham Heart Study. *The American journal of clinical nutrition* **2010**, *92*, 1165-1171, doi:10.3945/ajcn.2009.29106.
7. McKeown, N.M.; Yoshida, M.; Shea, M.K.; Jacques, P.F.; Lichtenstein, A.H.; Rogers, G.; Booth, S.L.; Saltzman, E. Whole-grain intake and cereal fiber are associated with lower abdominal adiposity in older adults. *The Journal of nutrition* **2009**, *139*, 1950-1955, doi:10.3945/jn.108.103762.
8. Liu, S.; Willett, W.C.; Manson, J.E.; Hu, F.B.; Rosner, B.; Colditz, G. Relation between changes in intakes of dietary fiber and grain products and changes in weight and development of obesity among middle-aged women. *Am J Clin Nutr* **2003**, *78*, 920-927, doi:10.1093/ajcn/78.5.920.
9. Lutsey, P.L.; Jacobs, D.R., Jr.; Kori, S.; Mayer-Davis, E.; Shea, S.; Steffen, L.M.; Szklo, M.; Tracy, R. Whole grain intake and its cross-sectional association with obesity, insulin resistance, inflammation, diabetes and subclinical CVD: The MESA Study. *Br J Nutr* **2007**, *98*, 397-405, doi:10.1017/s0007114507700715.
10. Esmaillzadeh, A.; Mirmiran, P.; Azizi, F. Whole-grain consumption and the metabolic syndrome: a favorable association in Tehranian adults. *Eur J Clin Nutr* **2005**, *59*, 353-362, doi:10.1038/sj.ejcn.1602080.
11. de la Fuente-Arrillaga, C.; Martinez-Gonzalez, M.A.; Zazpe, I.; Vazquez-Ruiz, Z.; Benito-Corchon, S.; Bes-Rastrollo, M. Glycemic load, glycemic index, bread and incidence of overweight/obesity in a Mediterranean cohort: the SUN project. *BMC Public Health* **2014**, *14*, 1091, doi:10.1186/1471-2458-14-1091.
12. Koh-Banerjee, P.; Franz, M.; Sampson, L.; Liu, S.; Jacobs, J.D.R.; Spiegelman, D.; Willett, W.; Rimm, E. Changes in whole-grain, bran, and cereal fiber consumption in relation to 8-y weight gain among men. *The American Journal of Clinical Nutrition* **2004**, *80*, 1237-1245, doi:10.1093/ajcn/80.5.1237.
13. Albertson, A.M.; Reicks, M.; Joshi, N.; Gugger, C.K. Whole grain consumption trends and associations with body weight measures in the United States: results from the cross sectional National Health and Nutrition Examination Survey 2001-2012. *Nutr J* **2016**, *15*, 8, doi:10.1186/s12937-016-0126-4.
14. Melanson, K.J.; Angelopoulos, T.J.; Nguyen, V.T.; Martini, M.; Zukley, L.; Lowndes, J.; Dube, T.J.; Fiutem, J.J.; Yount, B.W.; Rippe, J.M. Consumption of whole-grain cereals during weight loss: effects on dietary quality, dietary fiber, magnesium, vitamin B-6, and obesity. *J Am Diet Assoc* **2006**, *106*, 1380-1388; quiz 1389-1390, doi:10.1016/j.jada.2006.06.003.
15. Katcher, H.I.; Legro, R.S.; Kunselman, A.R.; Gillies, P.J.; Demers, L.M.; Bagshaw, D.M.; Kris-Etherton, P.M. The effects of a whole grain-enriched hypocaloric diet on cardiovascular disease risk factors in men and women with metabolic syndrome. *Am J Clin Nutr* **2008**, *87*, 79-90, doi:10.1093/ajcn/87.1.79.

16. Maki, K.C.; Beiseigel, J.M.; Jonnalagadda, S.S.; Gugger, C.K.; Reeves, M.S.; Farmer, M.V.; Kaden, V.N.; Rains, T.M. Whole-grain ready-to-eat oat cereal, as part of a dietary program for weight loss, reduces low-density lipoprotein cholesterol in adults with overweight and obesity more than a dietary program including low-fiber control foods. *J Am Diet Assoc* **2010**, *110*, 205-214, doi:10.1016/j.jada.2009.10.037.
17. Kristensen, M.; Pelletier, X.; Ross, A.B.; Thielecke, F. A High Rate of Non-Compliance Confounds the Study of Whole Grains and Weight Maintenance in a Randomised Intervention Trial-The Case for Greater Use of Dietary Biomarkers in Nutrition Intervention Studies. *Nutrients* **2017**, *9*, doi:10.3390/nu9010055.
18. Kristensen, M.; Toubro, S.; Jensen, M.G.; Ross, A.B.; Riboldi, G.; Petronio, M.; Bugel, S.; Tetens, I.; Astrup, A. Whole grain compared with refined wheat decreases the percentage of body fat following a 12-week, energy-restricted dietary intervention in postmenopausal women. *J Nutr* **2012**, *142*, 710-716, doi:10.3945/jn.111.142315.
19. Chang, H.C.; Huang, C.N.; Yeh, D.M.; Wang, S.J.; Peng, C.H.; Wang, C.J. Oat prevents obesity and abdominal fat distribution, and improves liver function in humans. *Plant Foods Hum Nutr* **2013**, *68*, 18-23, doi:10.1007/s11130-013-0336-2.
20. Harris Jackson, K.; West, S.G.; Vanden Heuvel, J.P.; Jonnalagadda, S.S.; Ross, A.B.; Hill, A.M.; Grieger, J.A.; Lemieux, S.K.; Kris-Etherton, P.M. Effects of whole and refined grains in a weight-loss diet on markers of metabolic syndrome in individuals with increased waist circumference: a randomized controlled-feeding trial. *Am J Clin Nutr* **2014**, *100*, 577-586, doi:10.3945/ajcn.113.078048.
21. Brownlee, I.A.; Moore, C.; Chatfield, M.; Richardson, D.P.; Ashby, P.; Kuznesof, S.A.; Jebb, S.A.; Seal, C.J. Markers of cardiovascular risk are not changed by increased whole-grain intake: the WHOLEheart study, a randomised, controlled dietary intervention. *Br J Nutr* **2010**, *104*, 125-134, doi:10.1017/s0007114510000644.
